# Editorial: Isocyanide-Based Multicomponent Reactions

**DOI:** 10.3389/fchem.2019.00918

**Published:** 2020-01-15

**Authors:** Jonathan G. Rudick, Shabnam Shaabani, Alexander Dömling

**Affiliations:** ^1^Department of Chemistry, Stony Brook University, Stony Brook, NY, United States; ^2^Department of Drug Design, University of Groningen, Groningen, Netherlands

**Keywords:** isocyanide chemistry, multicomponent reaction (MCR), diversity oriented synthesis, Ugi reaction, Passerini reaction, Groebke-Blackburn-Bienaymé reaction

Multicomponent reactions are an inspiring class of transformations in organic chemistry (Zarganes-Tzitzikas et al., 2015). These reactions, which incorporate three or more reactants into a single reaction product, offer advantages over traditional bimolecular reactions. Multicomponent reactions accelerate exploration of chemical space by reducing the number of synthetic and purification operations required to make a given target. The accompanying atom economy of multicomponent reactions further improve the sustainability of the chemical enterprise. The mechanisms of multicomponent reactions also challenge our understanding of subtle reactivity principles. Besides green chemistry attributes and mechanistic beauty, a key feature of multicomponent reactions that has not yet been fully embraced is the easy engineering of functional materials (Afshari and Shaabani, 2018). Functions can range from affinity ligands for immunoglobulin purification (Kruljec and Bratkovič, 2017) to imaging compounds in biological systems (Lin et al., 2017) to proteome-wide mapping of protein-protein interactions (Kambe et al., 2014) to molecular machines (García-González et al., 2018) to molecular keys for applications in advanced encryption standard cryptography with molecular steganography (Boukis et al., 2018). According to the basic principle *form follows function*, forms can be assembled in a unique fashion via multicomponent reactions from building blocks connected to certain properties. Such properties can be chirality, ligands for metals, fluorescence, extended π-systems with tunable HOMO–LUMO distances, or hydrogen bond donor-acceptor configurations. An emerging and rapidly growing field in this respect is the use of multicomponent reactions in polymer science and engineering (Llevot et al., 2017).

Among the most well-known and diverse class of multicomponent reactions are those in which an isocyanide (a.k.a., isonitrile) reagent is incorporated in the product. Isocyanide-based multicomponent reactions were some of the very first multicomponent reactions discovered in organic chemistry. Mario Passerini reported the reaction of an aryl isocyanide with ketones and carboxylic acids, the first isocyanide-based three-component reaction, nearly a century ago (Passerini, 1921). Ivar Ugi disclosed the first isocyanide-based four component reaction almost 40 years later (Ugi and Steinbrückner, 1960). Ugi's insights about the mechanisms of these multicomponent reactions have stimulated discoveries of numerous reaction variants. Ugi also recognized the potential for multicomponent reactions to enable combinatorial library synthesis and to serve as platforms for diversity-oriented synthesis. These pillars have sustained more than a half century of research into isocyanide-based multicomponent reactions. As we reflect upon the state-of-the-art in the field, these themes remain pervasive as synthetic organic chemists apply isocyanide-based multicomponent reactions to address challenges in biology (Neochoritis et al., 2019), polymers (Kakuchi, 2019), and materials science (Afshari and Shaabani, 2018).

Through continued method development and synergies with emerging technologies, the scope of isocyanide-based multicomponent reactions continues to expand. Several contributions to this themed collection highlight ongoing efforts to mine the richness and complexity of these reactions. Golantsov et al. report a novel reaction in which aryl(indol-3-yl)methylium tetrafluoroborates, aromatic isocyanides, and alcohols yield alky aryl(indol-3-yl)acetimidates. The indolium salts are derived from the condensation of *N*-alkyl indoles and aromatic aldehydes, which makes this a four-component reaction. Salvador and Andrade demonstrate dramatic improvements in efficiency and scalability of the Passerini three-component reaction involving arylglyoxals through adoption of the microwave-to-flow paradigm. Employing 1-unsubstituted 2-aminoimidazoles in the Groebke–Blackburn–Bienaymé reaction (Bienaymé and Bouzid, 1998; Blackburn, 1998; Groebke et al., 1998), Driowya et al. describe a novel method in which 1*H*-imidazo[1,2-*a*]imidazole-5-amines are prepared. Products of isocyanide-based multicomponent reactions, such as the classic Ugi reaction, are also versatile starting points for further diversification. Zidan et al. apply their recently developed “dianionic amide strategy” toward alkylation of Ugi reaction products.

Isocyanide-based multicomponent reactions have been highly successful in rapidly enumerating members of compound libraries, especially in drug discovery. Structural diversity in these libraries is critical to the elucidation of structure-activity relationships that drive development of new products and technologies. The emergence of resistance to drugs demands a deep cabinet of safe and effective drugs, in addition to a pipeline of new drug candidates. Derivatization of existing drugs represents one approach to generating libraries of compounds with high potential for translation to the clinic. Pedrola et al. exemplify this approach by derivatizing the antibiotic trimethoprim, an α-aminoazine, via the Groebke–Blackburn–Bienaymé reaction. Impressively, the resulting focused library contains compounds that are effective against methicillin-resistant *Staphylococcus aureus* (MRSA). Ochs et al. comprehensively investigate photoinduced electron transfer in a library of unimolecular exciplexes synthesized via the Ugi reaction.

Access to unexplored regions of chemical space is a pressing challenge for synthetic chemistry. Campaigns to discover biologically active compounds have saturated regions of chemical space identified from natural products and available in libraries of drug-like compounds. Judicious selection or design of the reactants in a given multicomponent reaction can afford previously unknown classes of compounds as products. Shaabani et al. provide such an example in their synthesis of oxazepine-quinazolone bis-heterocyclic scaffolds via an Ugi four-center three-component reaction. Yasaei et al. describe their discovery of an isocyanide-based multicomponent reaction that yields 5-(tosylquinolin-3-yl)oxazoles.

In conclusion, the past century has witnessed maturation of isocyanide-based multicomponent reactions from their seminal discovery to their present expansion into fields that rely upon chemical tools. Hallmarks of these reactions, such as sustainability and versatility, should motivate continued exploration of these powerful reactions as well as their adoption by researchers working beyond chemical synthesis. We hope that this themed collection helps to inspire readers embrace these opportunities in the century ahead.

## Author Contributions

All authors listed have made a substantial, direct and intellectual contribution to the work, and approved it for publication.

### Conflict of Interest

The authors declare that the research was conducted in the absence of any commercial or financial relationships that could be construed as a potential conflict of interest. The handling Editor declared a shared affiliation, though no other collaboration, with one of the authors JR.
